# Editorial on S.I. “Advances in Measuring Health and Wellbeing” in the *International Journal of Environmental Research and Public Health*

**DOI:** 10.3390/ijerph19095103

**Published:** 2022-04-22

**Authors:** Aurea Grané, Irene Albarrán

**Affiliations:** Statistics Department, Universidad Carlos III de Madrid, 28903 Getafe, Spain; irene.albarran@uc3m.es

Measuring the health and wellbeing of the population is the first step in visualizing the real needs of the population in order to promote healthy habits, as well as effective health and social policy responses.

Several goals of United Nations’ 2030 Agenda for Sustainable Development are related to reducing the risks of being unhealthy and socially vulnerable. Recently, we have experienced that, under the shock of a pandemic, these goals are scarcely fulfilled, even in developed countries.

This Special Issue contains 11 contributions from diverse research lines, representing different geographies, such as America, Asia or Europe. Some of them are related to elderly or disabled populations ([1,2,3,4]), while others present new financial or actuarial proposals ([5,6,7]) or focus on specific diseases, specific factors of mortality or on general wellbeing ([8,9,10,11]). All of them apply state-of-the-art tools or develop new methods to visualize and analyse data coming from multiple sources to produce reliable measurements on peoples’ health and wellbeing.

Irene Albarrán Lozano, Pablo J. Alonso-González and José Javier Núñez-Velázquez [1] estimate the years of residual life of the dependent population in Spain by applying a multi-state scheme. Specifically, they propose a methodology to estimate the probability of worsening the individual’s degree of dependency, that is, of modifying the life expectancy of an individual. This methodology is based on the relationship between overall mortality and the mortality of each group of interest and the health information contained in the macro-survey EDAD-2008.

Julia Córdoba and María José Bagnato [2] study the disabled population in Uruguay, aged between 18 and 64, whose health information is collected in the Survey of Social Protection (2016). The authors identify different groups characterised by different variables related to their health conditions, early dropouts from compulsory education, low levels of integration in the labour market, marked sense of loneliness and low levels of participation.

The study presented by Aurea Grané, Irene Albarrán and David E. Merchán [3] is focussed on the impact of a pandemic shock on the European population aged over 50 and aims to replicate COVID-19’s conditions. They use data from wave 7 of SHARE, before SHARE COVID-19 surveys were available. Their results suggest that the most affected European regions were those with a greater proportion of individuals initially deemed vulnerable in terms of mental and physical health, as well as countries where tourism and retail sectors were the most vital for their economies.

Mauricio Matus-López and Alexander Chaverri-Carvajal [4] present a comparison of the situation of long-term care services in five South American countries. The motivation for the work is based on the evolution of the population in the region, which tends to age and, as a consequence, has a greater potential demand for care services. This need for assistance in both instrumental and daily living activities is measured through a set of indicators.

J. Iñaki De La Peña, M. Cristina Fernández-Ramos and Asier Garayeta [5] study how to convert funds held in pension funds into funds dedicated to long-term care coverage. These authors analyse the conditions that must be met to achieve such conversion without increasing the costs of a private pension scheme and adapting the amounts to be received to the individual’s life expectancy.

Ana Debón, Steven Haberman, Francisco Montes and Eduardo Otranto [6] evaluate the use of different mortality models on a set of indicators. All models are variants of the original Lee–Carter model. The results obtained are compared in terms of both probabilities and values achieved for the mortality indicators used.

The work by Pu Liao, Zhihong Dou and Xingxing Guo [7] is focused on the impact of parental health shocks on children’s education. They analyse the situation in China and the role of health insurance in absorbing these effects. Given China’s economic and social structure, the authors suggest that the effect of the impact of fathers’ health on children is greater than that of the mothers.

Iwona Bonikowska, Katarzyna Szwamel and Izabella Uchmanowicz [8] investigate the effects of factors such as the acceptance of a disease and clinical circumstances on adherence to recommended treatments. They study a sample of 200 patients affected by type 2 diabetes mellitus. After using a one-way ANOVA, the results indicate that both the average age and the number of daily pills are significant statistically different between adhering and non-adhering patients.

Federica Cugnata, Silvia Salini and Elena Siletti [9] use a Bayesian framework to study the link that might exist between traditional statistics and the information collected by social networks. The study collects Italian data from the period 2012 to 2017 from Twitter and ISTAT, and the results suggest that the information included in the big data anticipates the information that will later be collected in official statistics.

Martínez-Urbistondo et al. [10] study the impact that certain habits have on the quality of physical and mental health. Specifically, they analyse the effects of tobacco consumption, dietary intake of ultra-processed pastries, carbonated drinks, sleep time and physical activity and demonstrate the relationship between these unhealthy habits and lower levels of the QoL index.

Finally, James Ming Chen, Mira Zovko, Nika Šimurina and Vatroslav Zovko [11] focus their study on the effects of PM2.5 pollution on the mortality of the population of 27 countries of the European Union. They assess the success (or failure) of PM2.5 pollution management through a collection of statistical techniques ranging from linear regression models to machine learning. The authors give an additional interpretation of the variables by using PCA.
